# Quantitative analysis of co-oligomer formation by amyloid-beta peptide isoforms

**DOI:** 10.1038/srep28658

**Published:** 2016-06-27

**Authors:** Marija Iljina, Gonzalo A. Garcia, Alexander J. Dear, Jennie Flint, Priyanka Narayan, Thomas C. T. Michaels, Christopher M. Dobson, Daan Frenkel, Tuomas P. J. Knowles, David Klenerman

**Affiliations:** 1 Department of Chemistry, University of Cambridge, Lensfield Road, Cambridge CB2 1EW, UK.

## Abstract

Multiple isoforms of aggregation-prone proteins are present under physiological conditions and have the propensity to assemble into co-oligomers with different properties from self-oligomers, but this process has not been quantitatively studied to date. We have investigated the amyloid-β (Aβ) peptide, associated with Alzheimer’s disease, and the aggregation of its two major isoforms, Aβ40 and Aβ42, using a statistical mechanical modelling approach in combination with *in vitro* single-molecule fluorescence measurements. We find that at low concentrations of Aβ, corresponding to its physiological abundance, there is little free energy penalty in forming co-oligomers, suggesting that the formation of both self-oligomers and co-oligomers is possible under these conditions. Our model is used to predict the oligomer concentration and size at physiological concentrations of Aβ and suggests the mechanisms by which the ratio of Aβ42 to Aβ40 can affect cell toxicity. An increased ratio of Aβ42 to Aβ40 raises the fraction of oligomers containing Aβ42, which can increase the hydrophobicity of the oligomers and thus promote deleterious binding to the cell membrane and increase neuronal damage. Our results suggest that co-oligomers are a common form of aggregate when Aβ isoforms are present in solution and may potentially play a significant role in Alzheimer’s disease.

Neurodegenerative diseases, such as Alzheimer’s disease (AD), are devastating and incurable conditions associated with the misfolding and aggregation of native monomeric proteins^1^. The deposition of aggregated amyloid-β peptide (Aβ) in the brain is a pathological hallmark of AD^2^. Aβ is formed from the cleavage of a transmembrane receptor, the amyloid precursor protein (APP), in various locations to generate peptides of varying lengths, most commonly 40 and 42 residues (Aβ40 and Aβ42)^3^. The Aβ42 isoform has an additional Ile-Ala dipeptide at its C terminus making it more hydrophobic and more aggregation-prone than Aβ40^4^,^5^. Hence, while the relative ratio of the Aβ40 to Aβ42 in cerebrospinal fluid (CSF) is approximately 9:1, the amount of Aβ42 is enriched relative to Aβ40 in deposits such as amyloid plaques^6^,^7^. Moreover, some early-onset versions of AD have been related to the overproduction of Aβ42 relative to Aβ40^8^, and an increase in the ratio of Aβ42 to Aβ40 cleaved from APP has been correlated to increases in toxicity both *in vitro* and *in vivo*^9^,^10^,^11^,^12^,^13^,^14^.

Although solid fibrillar deposits of Aβ accumulate in AD brains, the major cytotoxic effects causing the earliest pathological events are associated with smaller aggregates, Aβ oligomers^15^. Such species are formed via the association of monomeric Aβ and ultimately polymerize into amyloid fibrils when the total protein concentration exceeds the critical aggregation concentration (CAC)^16^. Due to their transient presence and low abundance, the oligomers have been difficult to characterise using conventional experimental techniques^17^, particularly in the systems containing multiple isoforms of Aβ. There have been numerous studies of the mixtures of Aβ isoforms, demonstrating that Aβ40 and Aβ42 co-interact during the aggregation reaction^18^,^19^,^20^,^21^,^22^. Furthermore, there is evidence that Aβ40 and Aβ42 can form co-oligomers *in vitro*^10^,^18^, and on the surface of neurons^23^. A detailed study revealed that while Aβ40 and Aβ42 form separate fibrils in solution, the peptides co-interact in the early stages of Aβ aggregation, during primary nucleation^24^.

In these previous studies, it has not been possible to determine the concentration and composition of the formed self- or co-oligomeric species of Aβ40 and Aβ42. Moreover, since most biophysical studies are typically performed at non-physiological high-micromolar concentrations of Aβ, it has not been possible to extrapolate the observations to very low total concentrations of Aβ peptide observed *in vivo*^25^. Because of the demonstrated strong and non-linear concentration dependence of Aβ aggregation^26^,^27^,^28^,^29^, a meaningful extrapolation would require direct measurements of Aβ oligomer populations at sub-micromolar peptide concentrations. In order to address this, we combine here direct single-molecule measurements of oligomer populations at low Aβ concentrations with a statistical mechanical model to estimate the number and composition of the oligomers present under equilibrium conditions, and subsequently investigate how changing the ratios of the two Aβ isoforms affects the resulting oligomer populations.

## Results and Discussion

### Modelling approach

In this study, the relevant thermodynamic parameter characterising oligomerization is the free energy of monomer addition, Δ*G*°, independent of oligomer size, and this single parameter forms the basis for our model, as described in detail in Supplementary Information.

**Figure 1:**
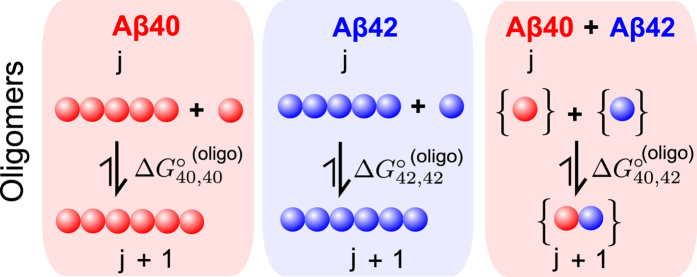
Schematic of the statistical mechanical model used to estimate Aβ oligomer numbers and relative composition. For the single-species datasets, the model considers oligomers of any length, whereas for the co-oligomerising datasets it considers monomers and dimers as the single-species analysis predicts a very low number of oligomers larger than dimers (Fig. 3b).

In the model we consider the major contribution to the energetics of the oligomeric aggregates to emerge from nearest neighbour interactions. We thus treat self-oligomers as simple non-interacting one-dimensional chain structures with nearest-neighbour interactions independent of the chain length (Fig. 1). We note that the assumption of one-dimensional chain structures is not restrictive under our experimental conditions, where both self- and mixed oligomers can be inferred by our self-oligomer model to be predominantly dimeric (see Supplementary Information), and therefore larger non-linear structures where geometric effects can play a major role are not expected to perturb the analysis. This result permits us to formulate and employ a simple model for co-oligomers, containing monomers of both Aβ40 and Aβ42, which considers only dimers. The system behaviour is thus effectively governed by the Gibbs free energy Δ*G*° released upon adding two monomers together to form a new intermolecular interaction. Note that while the assumption of the size-independent binding free energy is valid for the studied Aβ system, it is not applicable to non-filamentous growth assemblies.

In general for a linear aggregation process, we can identify 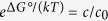 with a CAC *c* for a standard concentration *c*_0_ of 1 M^30^. The nature of the species present at equilibrium depends strongly on the initial concentration of the monomeric peptides. When the monomer concentration is below the CAC, the majority of the peptides in the system are in their monomeric states and only a few aggregates are formed consisting of a small number of monomers. By contrast, above the CAC, most molecules are present as aggregates. These aggregates are either oligomers or fibrils. Previous single-molecule^31^ and bulk data^32^,^33^,^34^ indicate that these two species differ in their structures, and thus we allow for separate Δ*G*° for the oligomeric and fibrillar states, Δ*G*°^(*oligo*)^ and Δ*G*°^(*fib*)^. Moreover, oligomers are populated only for small aggregation numbers, while mature fibrils are observed for sizes that exceed 1000 monomers^35^. At low concentrations, therefore, below the CAC, the formation of large aggregates is suppressed, and the majority of aggregates are oligomeric. When the total concentration reaches the CAC, the majority of monomers are sequestered into fibrillar forms, and the concentration of oligomers does not increase even when the total peptide concentration is increased. Thus we expect the initial increase in aggregate concentration to be controlled by the free energy of oligomer formation 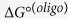. Once the total peptide concentration reaches the CAC, 
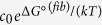
, the theory predicts a plateau in the concentration of oligomers, controlled by the free energy of fibril formation 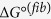. A recent study shows that the formation of self-fibrils of the Aβ isoforms is favoured *in vitro*^24^, which implies that there is a significant difference between the 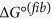 when adding a monomer to a self-fibril or a fibril of different composition for Aβ40 and Aβ42. However, the same study suggests that the difference is smaller for 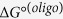 when adding a monomer to a self- or mixed oligomer.

### Single-molecule measurements

Having established the described theoretical approach, we then used single-molecule two-colour coincidence detection (TCCD)^36^ in order to measure directly the concentration of Aβ oligomers present in solutions below and around the CAC, for Aβ40, Aβ42 and a 1:1 mixture of Aβ40 and Aβ42 (see Supplementary Information for detailed methods). Prior to the measurements, it was confirmed that the fluorescently labelled Aβ used in these experiments was able to self-assemble into amyloid fibrils (Supplementary Fig. S1), in agreement with our previous results^35^, and with multiple other studies using the same fluorescent peptides^23^,^37^,^38^,^39^.

Initially, we measured the fibril CAC through two independent methods: firstly by determining the concentration of soluble species in equilibrium with fibrils, which coincides with the fibril CAC at high concentration, above the fibril CAC^30^ (Supplementary sections S1.4 and S2.2). Secondly, we determined the concentration at which the oligomer concentration ceases to increase with total peptide concentration and reaches a plateau phase (Supplementary sections S1.3 and S2.1); the theory predicts that this transition should take place at the fibril CAC.

The total concentration of the released species in the former approach, which corresponds to the CAC, was measured to be 94 ± 37 nM for Aβ40, and 28 ± 4 nM for Aβ42 at pH 7.4. The value for Aβ40 is in good agreement with the previous result of 100 nM at pH 7.4^40^, and the value for Aβ42 is lower than a previously reported value of 0.2 μM at pH 8^41^, consistent with a reported decrease in CAC with lowering the pH^40^. This gives values of the free energy for fibril formation as 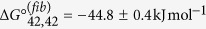 for Aβ42 and 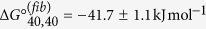 for Aβ40. The result for Aβ40 is within the range of previously reported values for the unlabelled peptide^42^,^43^, which were −37.7 kJ mol^−1^ and −46.7 kJ mol^−1^, indicating that the presence of the fluorophore labels at the N-terminus does not substantially alter the free energy of fibril formation. The observation that Aβ42 fibrils disaggregate to a lesser extent than fibrils of Aβ40 suggests that the Aβ42 fibrils are more stable than their Aβ40 counterparts, correlating well with previous reports of Aβ disassembly and stability^44^,^45^.

**Figure 2:**
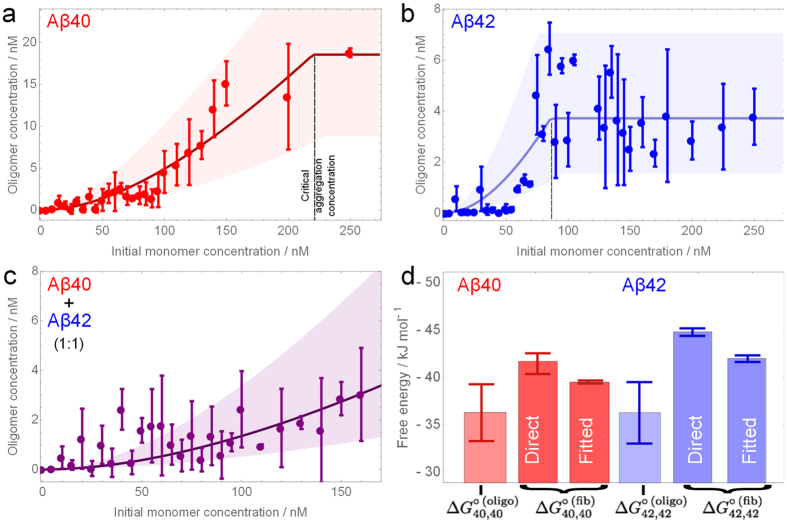
Equilibrium oligomer concentrations as a function of the total initial monomer concentration in the aggregation reaction. (Error bars SD, N (samples) = 3). The oligomer concentration was modelled and fitted separately for both Aβ40 **(a)**, Aβ42 **(b)**, and the 1:1 mixture **(c)**; allowing extraction of the free energies of oligomerization and estimation of the CAC for Aβ40 and Aβ42 (fitted curves shown overlaid). The shaded bounds on these charts are curves plotted using the maximum and minimum free energies of oligomerization, and of fibril formation (given by the CAC) that still lie within the majority of the error bars. **(d)** The fitted free energies of oligomerization are also shown in comparison to the free energies of fibril formation obtained by direct measurement of the CAC (“Direct”), and also the free energies of fibril formation obtained from the fitted estimation of the CAC (“Fitted”).

Next, we combined equal quantities of monomeric peptide singly labelled with a blue-fluorophore with monomeric peptides singly labelled with a red-fluorophore, using low concentrations of total Aβ, 1–250 nM (Supplementary section S1.1). The solutions were left for 72 hours at 37 °C until a steady-state population of oligomers and monomers was generated in each case. We verified that the populations of oligomers did not change upon incubation for up to 7 days (Supplementary Fig. S2) confirming the attainment of the steady-state past 72 hours. Given the chosen restriction of our incubations to up to 7 days, we do not exclude the possibility that the system could undergo further changes at longer time-scales. As the monomeric peptides self-associate to generate oligomers, we can distinguish them from monomers by the criteria of coincidence and quantify the oligomeric populations by TCCD (Supplementary section S2.1). The results are shown in Fig. 2, and the oligomer concentrations are in the range of 0–20 nM for Aβ40, 0–4 nM for Aβ42 and, strikingly, around 0–3 nM for mixed Aβ40-Aβ42 species. We confirmed in a series of control experiments that the monitored signal arises from the interactions between the peptides and not from random association of the fluorescent probes, as detailed in Supplementary section S2.1. The error bars are relatively high in these experiments due to the low oligomer concentrations and inherent sample to sample variations. However, the results appear to follow the prediction from the theory and allow an estimate of the 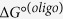 values to be obtained in each case, as is described below.

### Estimations of the free energies of oligomer and fibril formation

From the results in Fig. 2a,b, the similarity in the slopes of the growth regions below the CAC of the Aβ40 and Aβ42 self-oligomerizing systems suggests that there is no large difference in the mean free energy of oligomerization in both cases. By fitting our model to the self-oligomerizing systems (Supplementary section S6), we estimate the free energy of oligomerization for Aβ40, 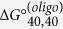, as −36.3 ± 3.0 kJ mol^−1^, and similarly 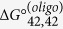 for Aβ42 as −36.3 ± 3.2 kJ mol^−1^ (Fig. 2). These values are different from those for the fibrils, which is consistent with the expected differences in the structure of oligomers and fibrils. The CAC for Aβ40 is estimated as 222 ± 10 nM by the same fitting procedure, and the CAC for Aβ42 is estimated as 86 ± 10 nM; these values allow independent estimation of 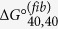 as −39.5 ± 0.1 kJ mol^−1^ and 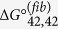 as −42.0 ± 0.3 kJ mol^−1^, demonstrating broad consistency with the direct measurements. The value of 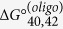 is estimated to be −32.6 ± 2.6 kJ mol^−1^ (Supplementary section S7), and the absence of apparent plateau in the co-oligomer plot (Fig. 2c) is consistent with both isoforms being present below their CAC values. According to these results, summarized in Fig. 2d, in all cases the free energy of oligomerization is large and negative. The seemingly small difference in the free energy of oligomerization for the formation of co-oligomers in comparison to the self-oligomers, however, leads to lower abundance of these species, as will be described later. To point out, while there have been previous reports of the free energy for fibril formation of Aβ^42^,^43^ and other amyloidogenic proteins^46^, the directly measured free energies of oligomerization for Aβ40, Aβ42 and Aβ40-Aβ42, to our knowledge, are reported for the first time. The formation of the spectator co-oligomers means that, in the presence of both Aβ40 and Aβ42, fewer self-oligomers of Aβ40 or Aβ42 will be formed, so growth into Aβ40 or Aβ42 fibrils may be suppressed. This may provide an explanation of why the aggregation kinetics of both isoforms were observed to be mutually affected in the previous related studies^10^,^18^.

### Predictions of oligomer populations at 1 nM concentration of Aβ

**Figure 3:**
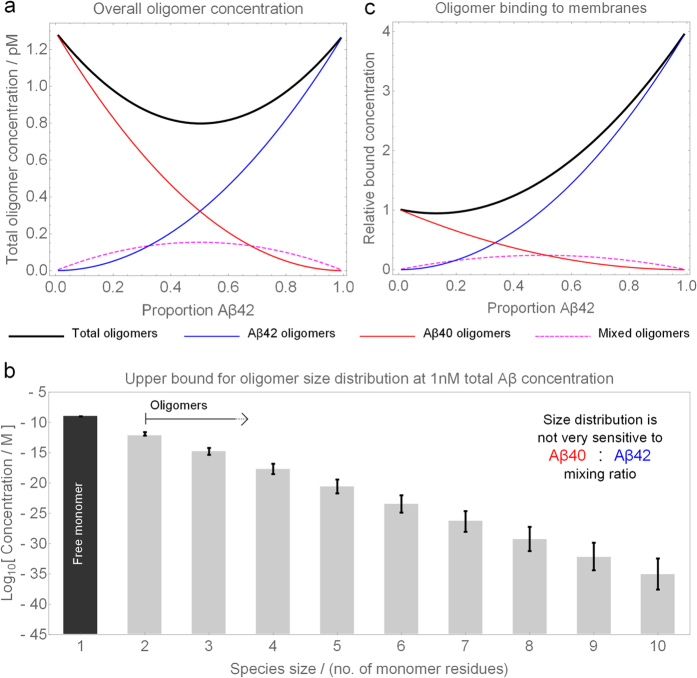
Simulation of Aβ40-Aβ42 co-oligomerization equilibrium behaviour at a total Aβ concentration of 1 nM for a range of Aβ42 proportions, using 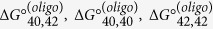. Simulations at 5 nM and 10 nM of total Aβ are shown in Supplementary Fig. S3. **(a)** Total oligomer concentration and composition as a function of Aβ42 proportion. **(b)** Estimated concentrations of oligomers of different sizes at 1 nM total protein concentration, calculated by assuming that 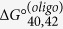 is unchanged from the single-species value (in which case the ratio of Aβ40:Aβ42 is irrelevant). The true distribution will decline with oligomer size even more rapidly, as visual inspection of the data shows 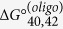 to be less favourable than the single-species values. The error bars correspond to averaged uncertainty in the Δ*G* measurements. **(c)** The relative concentration of oligomers estimated to be bound to the surface of a neuronal cell, expressed relative to the concentration of oligomers bound to the surface at 1 nM of Aβ40. This result assumes that the relative affinity of co-oligomers for the cell membrane is 2 times higher than the affinity of Aβ40 oligomers, and that the relative affinity of Aβ42 oligomers is 4 times higher than that of Aβ40 oligomers.

The obtained experimental values for the free energies of oligomerization can be used to predict the total oligomer concentration and the fraction of mixed and self-oligomers at pre-defined Aβ concentrations and ratios of Aβ40 and Aβ42. The measurements in this study have been carried out at 0–250 nM starting concentrations of Aβ, the range which is substantially lower than what can be accessed using more conventional experimental methods^17^. However, it is known that the physiologically related total concentration of this peptide is in the range of 1–10 nM^25^. To infer the information about oligomer types and sizes at these extremely low concentrations of Aβ, we can use the derived free energy values and set the starting total Aβ concentration to a chosen value within the physiological range. Figure 3a shows how the distributions of oligomer sub-populations are predicted to change in Aβ40 and Aβ42 mixture as a function of the Aβ42 proportion, when the total Aβ concentration is chosen to be 1 nM. Similar predictions with the total concentrations set to 5 nM and 10 nM are shown in Supplementary Fig. S3. Due to less negative free energy of co-oligomerization, the resulting predicted co-oligomer populations are lower than the self-oligomer populations at all mixing ratios of Aβ40 and Aβ42. The predominant oligomers at a physiologically-relevant ratio of 9:1 of Aβ40 to Aβ42 will be the oligomers of Aβ40, then a small fraction of co-oligomers with only a tiny fraction of Aβ42 oligomers. Moreover, the size distributions can be also inferred, as shown in Fig. 3b. At 1 nM of the total protein concentration, the main oligomers present are dimers, and the number of oligomers is predicted to decrease exponentially with oligomer size.

Since Aβ42 peptide is more hydrophobic than Aβ40, it is plausible that this difference would be conserved in the derived oligomers, which could influence their properties. Our previous study suggested that Aβ40 and Aβ42 oligomers are both cytotoxic, once formed^47^. Furthermore, our previous experimental data on the binding of Aβ40 and Aβ42 oligomers to neuronal cells suggested that, at the lowest concentration measured, the relative affinity of Aβ42 oligomers for the cell membrane was 4 times that of the Aβ40 oligomers^48^. If we assume that the affinity of the co-oligomers is 2 times that of the Aβ40 oligomers, a value intermediate between Aβ40 and Aβ42 oligomers, and that the majority of oligomers are dimers, according to Fig. 3b, we can then predict how the relative concentration of membrane-bound oligomers varies as a function of Aβ42 proportion, as is presented in Fig. 3c. This analysis predicts a clear increase in the relative number of oligomers bound to the cell surface with the increase in the proportion of Aβ42. Interestingly, the minimum number of cell-bound oligomers in this simulation occurs at a ratio of 9:1 of Aβ40 to Aβ42. Note that the oligomer size distribution (Fig. 3b) is not significantly altered by the ratios of Aβ40 and Aβ42 since the free energies of oligomerization are all comparable and in all cases are dominated by dimers. However, more of these dimers will contain Aβ42 as the proportion of Aβ42 increases. We note that while our analysis in Fig. 3c considers dimers, as they are the most abundant oligomers in our system, the prediction of absolute concentrations of large surface-bound oligomers is beyond the scope of this analysis due to the absence of additional oligomer to membrane interactions.

Clearly, this model may not be fully applicable to the Aβ oligomers in AD, since their formation under more complex *in vivo* environment is potentially affected by numerous extrinsic factors such as, for instance, the presence of small molecules and proteins, lipid surfaces, altered pH or ionic strength and the underlying assumption of thermodynamic equilibrium may not be correct. Nevertheless, it is interesting to compare the predictions of our model to what is actually observed in humans. From the results of a previous quantitative study where stable synthetic Aβ dimers were used as standards, the concentrations of Aβ oligomers in CSF of AD patients and controls were identified to be in the sub-picomolar range, in agreement with our predictions of the oligomer concentration at a total Aβ concentration of 1 nM, although the low concentration prevented the determination of the oligomer sizes in that work^49^. It is also interesting that the oligomer concentration measured *in vivo* appears to be determined by the Aβ monomer concentration in the CSF. AD patients will also have amyloid plaques containing Aβ40 and predominantly Aβ42 fibrils. In our experiments, the oligomer concentrations above fibrils are those shown in the plateau regions in Fig. 2. Overall the total oligomer concentration is about 20 nM, which is two orders of magnitude larger than around 0.1 pM observed *in vivo*^49^. This suggests that either the exchange between oligomers and fibrillar plaques does not occur to any significant extent *in vivo*, or that there are additional contributing factors which are not present in our analysis, for example, active degradation mechanisms that remove oligomers^50^. To note, even though the amount of Aβ42 in the CSF is generally observed to decrease in AD, our model would predict that this has little effect on the total oligomer concentration, because their population is largely dominated by Aβ40 oligomers. This may provide a simple explanation for why most diagnostic tests for AD to date based on detecting the Aβ oligomer concentration in CSF observe little significant difference between controls and AD patients^51^.

Our model can be applied to predict how the number of membrane-bound oligomers changes upon increasing the ratio of Aβ42 to Aβ40 using pre-defined concentrations of Aβ which correlate with the onset of AD. While this analysis does not take account of any additional factors that may contribute to the disease in man^2^, it serves to illustrate how significantly the starting concentrations of the two isoforms influence the resulting populations of potentially pathogenic oligomers. For example, in the case of the Beyreuther/Iberian mutation^52^,^53^ where the ratio of Aβ42 to Aβ40 is as high as 22:1^54^, early onset of AD occurs before 40 years of age. If we use a starting peptide ratio of 22:1 in our simulations, the number of oligomers on the cell surface is predicted to increase by a factor of 4 relative to Aβ40 self-oligomers. Not only can a raised proportion of Aβ42 be pathogenic *in vivo*, but also the overall overproduction of Aβ. For example, in Down’s syndrome there is an extra copy of the gene for APP, meaning that the total Aβ concentration is elevated by a factor of 1.5, leading to an early-onset AD at around 40 years^55^. If we increase the total peptide concentration by a factor of 1.5 in our model, the total Aβ oligomer concentration increases by 125%, and the predicted number of cell-bound oligomers increases by a factor of 2.1 relative to the number of oligomers bound for 100% Aβ40 at the initial total Aβ concentration. While a change in Aβ40 to Aβ42 ratio from 9:1 to 7:3 results in no overall increase in the total number of oligomers, there is a significant difference in their predicted composition, with more co-oligomers being bound. In addition, the co-oligomers may be more persistent than self-oligomers, since they cannot grow into less toxic fibrils^24^, so it is possible that the increased persistency of co-oligomers additionally contributes to the increased toxicity.

## Summary and Conclusions

Our results show that co-oligomers of Aβ40 and Aβ42 can be formed at sub-micromolar concentrations of Aβ with little free energy penalty. This finding can be rationalized if there is little change in the free energy of oligomerization due to the additional Ile-Ala dipeptide on Aβ42, suggesting that the environment of these additional dipeptides does not change significantly between the monomeric and oligomeric state, and that the contribution to the free energy of oligomerization from the formation of contacts between other amino acids dominates the energetics relative to the role of the additional two residues at the C-terminus. There are multiple other isoforms of Aβ present because of truncations, mutations, ubiquitination or post-translational modifications. If there is no high penalty in the free energy of co-oligomerization, then these species may potentially be formed by various isoforms of the peptide since mixing entropy under such conditions favours the formation of mixed rather than purely segregated aggregates. It is likely therefore that under *in vivo* conditions where multiple isoforms are present, such mixed aggregates are prevalent. Thus, any comprehensive therapeutic strategy based on antibodies that bind Aβ may need to take account of the presence of co-oligomers in addition to self-oligomers of Aβ. Therefore, it can be envisaged that in many situations both co-oligomers could be formed, which have the propensity to be more toxic due to their longer persistence time, as well as self-oligomers, which might be effective seeds and may cause prion-like spreading^56^. At present it is still unclear which forms of Aβ are the true pathogens in AD^57^, and the contribution of Aβ co-oligomers to AD may not have been recognized to date.

## Methods

### Aβ peptide stock preparation

Monomeric solutions of HiLyteFluor 488 and HiLyteFluor 647-labelled Aβ40 and Aβ42 (Anaspec, Fremont) were prepared as described previously^35^,^58^, by dissolving the lyophilized peptide in NaOH, pH 12, sonication over ice for 25 min (Bandelin Sonorex), and flash-freezing into aliquots and storage at −80 °C. Initially, stock solutions were prepared by diluting the protein solutions into SSPE buffer (150 mM NaCl, 10 mM Na_2_H_2_PO_4_ x H2O, 10 mM Na_2_EDTA, 0.01% NaN_3_, pH 7.4) followed by serial dilutions with the same SSPE buffer, pH 7.4, to the desired aggregation reaction concentrations. Prior to the experiments, the ability of the labelled peptides to self-assemble into amyloid fibrils at pH 7.4 was confirmed by Transmission Electron Microscopy (TEM) imaging (Supplementary Fig. S1), and was in agreement with our previous control experiments using identical peptide preparations^35^,^48^.

### Αβ Oligomer Preparation

For the incubations, 1:1 molar ratios of 488 and 647-labelled samples were used, either 488:647 Aβ40 or 488:647 Aβ42 for the self-aggregations, or 488 Aβ40:647 Aβ42 for the mixed aggregations. Three separate samples for each concentration (0–250 nM of total Aβ) and protein combination were prepared. LoBind microcentrifuge test-tubes (Eppendorf, Hamburg, Germany) were used for all incubations to prevent surface absorption, as was found to be effective in our previous studies^59^,^60^. Incubations were performed for 3 d at 37 °C with rotary shaking (200 rpm, New Brunswick Scientific Innova), and subsequently analysed using single-molecule two-colour coincidence detection (TCCD). This time period was found optimal, as the observed levels of aggregates did not change upon longer incubations (7 d), as shown in Supplementary Fig. S2.

### CAC Sample Preparation

For the critical aggregation concentration (CAC) measurements using fibril disaggregation, fibrils were first prepared by 72-hour incubation of 10 μM solutions of singly-labelled protein samples, either 488 Aβ40, or 647 Aβ40, and 10 μM 488 Aβ42 or 647 Aβ42, pH-adjusted to 7.4 and incubated under the same conditions as above. Pelleting was carried out by centrifugation at 12,800 × g for 15 min, followed by two identical washing steps involving removal of the supernatant, washing of the pellet and additional centrifugation for 5 min. Finally, the pellet was re-suspended in fresh pH 7.4 SSPE buffer, by adding 100 μL buffer to ensure the excess of fibrillar material. The resulting samples were incubated under quiescent conditions at 37 °C for 3 d, and centrifuged for 15 min at 12,800 × g prior to measurements. For the confirmation of equilibrium past 3 d, identical samples were incubated for longer (7 d), yielding agreeable results.

### Measurements of Αβ Oligomers

Two-colour coincidence detection (TCCD) with dual excitation in 488/633 mode was performed using single-molecule confocal instrument and methodology as previously described in detail^35^, utilizing a detection under fast-flow, as described before^59^. Briefly, this method uses two overlapped lasers of different wavelengths in order to distinguish between species bearing two different fluorophores and singly-labelled species using the criteria of temporal coincidence^35^. Aggregates bearing two different fluorophores will produce fluorescent signals of two different colours that are coincident in time, while singly labelled monomers will produce non-coincident bursts. Full details of the experimental protocol and data analysis are in Supplementary section S2.

### CAC Measurements and Analysis

These measurements were performed to determine the total concentration of Aβ, released from the Aβ fibrils into buffer solution upon prolonged incubations, similarly to previously described methods^35^. This was done by relating the burst counts of the measured soluble supernatants to the burst counts from a DNA standard of precisely known concentration, as detailed in Supplementary section S2.

## Additional Information

**How to cite this article**: Iljina, M. *et al*. Quantitative analysis of co-oligomer formation by amyloid-beta peptide isoforms. *Sci. Rep*. **6**, 28658; doi: 10.1038/srep28658 (2016).

## Supplementary Material

Supplementary Information
