# Supplementary material for: Quantitative analysis of co-oligomer formation by amyloid-beta peptide isoforms
Source: Sci Rep. 2016 Jun 27;6:28658. doi: 10.1038/srep28658 (PMC4921824; doi:10.1038/srep28658)
Supplement: Supplementary Information [file srep28658-s1.pdf]

Supplementary Information

Quantitative analysis of co-oligomer formation by amyloid-beta peptide

Marija Iljina*, Gonzalo A. Garcia*, Alexander J. Dear*, Jennie Flint*,
Priyanka Narayan, Thomas C. T. Michaels, Christopher M. Dobson,
Daan Frenkel, Tuomas P. J. Knowles** and David Klenerman**

**Equally contributing authors.*

*** Correspondence may be addressed to: tpjk2@cam.ac.uk, dk10012@cam.ac.uk*

S1. ADDITIONAL CONTROLS FOR Aβ SAMPLE PREPARATION

S1.1. TEM Imaging

1:1 488:647 Hilyte Fluor Aβ40 or Aβ42 peptide solutions were prepared at 10 μM concentration and incubated for 3 d at 37 °C with rotary shaking (200 rpm, New Brunswick Scientific Innova). 10-15 μL volumes of the samples were applied onto carbon-coated 400-mesh copper grids (EM Resolutions) for 5 min, and washed with distilled water. Negative staining was carried out by using 2 % (w/v) uranyl acetate. TEM images were acquired using Tecnai G2 microscope (13218, EDAX, AMETEK) operating at an excitation voltage of 200 kV. Representative results are shown in Figure S1, and confirm the ability of the fluorescently-labeled peptides to assemble into amyloid fibrils.

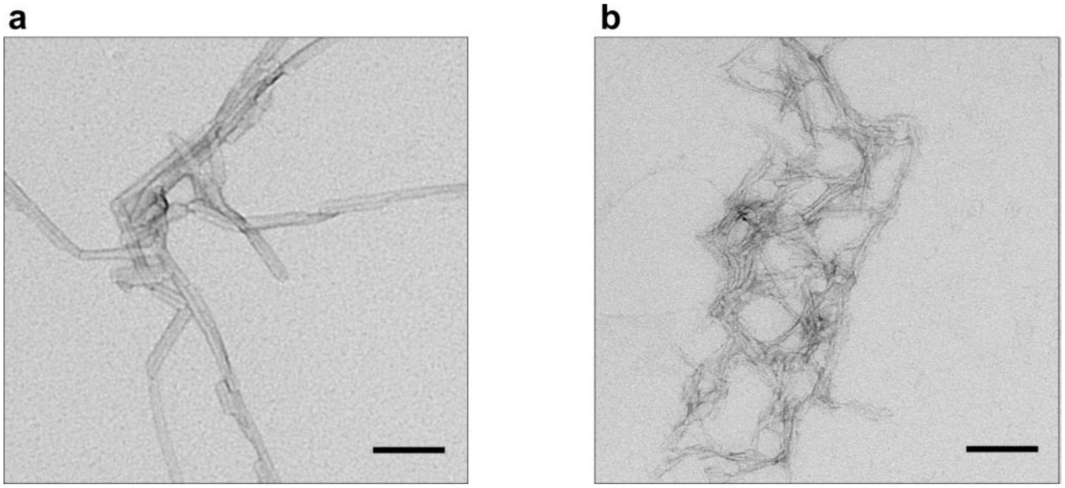

Figure S1. TEM images of amyloid fibrils formed by 1:1 488:647 Hilyte Fluor labeled Aβ40 (**a**) and 1:1 488:647 Hilyte Fluor labeled Aβ42 (**b**). Scale bar is 100 nm.

S2. TCCD DATA ACQUISITION AND ANALYSIS

S2.1. Measurements of Aβ Oligomers

For the measurements, dual-labeled protein samples were diluted in SSPE buffer up to 100 pM concentration suitable for the single-molecule analysis, immediately introduced into a straight channel of a microfluidic device via a gel-loading tip (200 μL, Life technologies) and withdrawn at a constant rate of 1 cm s^{-1} via a syringe pump (PHD2000, Harvard Apparatus). Overlapped laser beams were focused into the middle of the channel. For each sample, data were acquired for 600 s, with 100 μs bin-width, chosen according to the expected residence time in the excitation region at the chosen flow speed,[2] 100,000 bins per frame and a total of 60 frames. The fluorescence photon traces in two separate channels, the emission from 488 (blue) and 647 (red) dyes, were collected simultaneously and outputted using custom-programmed field-programmable gate array (FPGA, Colexica). All measurements were made at ambient temperature around 20 °C.

The collected photon traces were analyzed using custom-written Igor Pro 6.22 (Wavemetrics) software similarly to previously described.[1] The data were corrected for autofluorescence in the blue (1.86 photons bin^{-1}) and red (1.33 photons bin^{-1}) channels and for the crosstalk (3% from blue to red and negligible in the opposite direction). Photon bursts with intensities greater than the threshold of 15 photons bin^{-1} in the blue, and in the red channels, were selected according to previously established threshold selection approach that allows maximizing the detection of co-

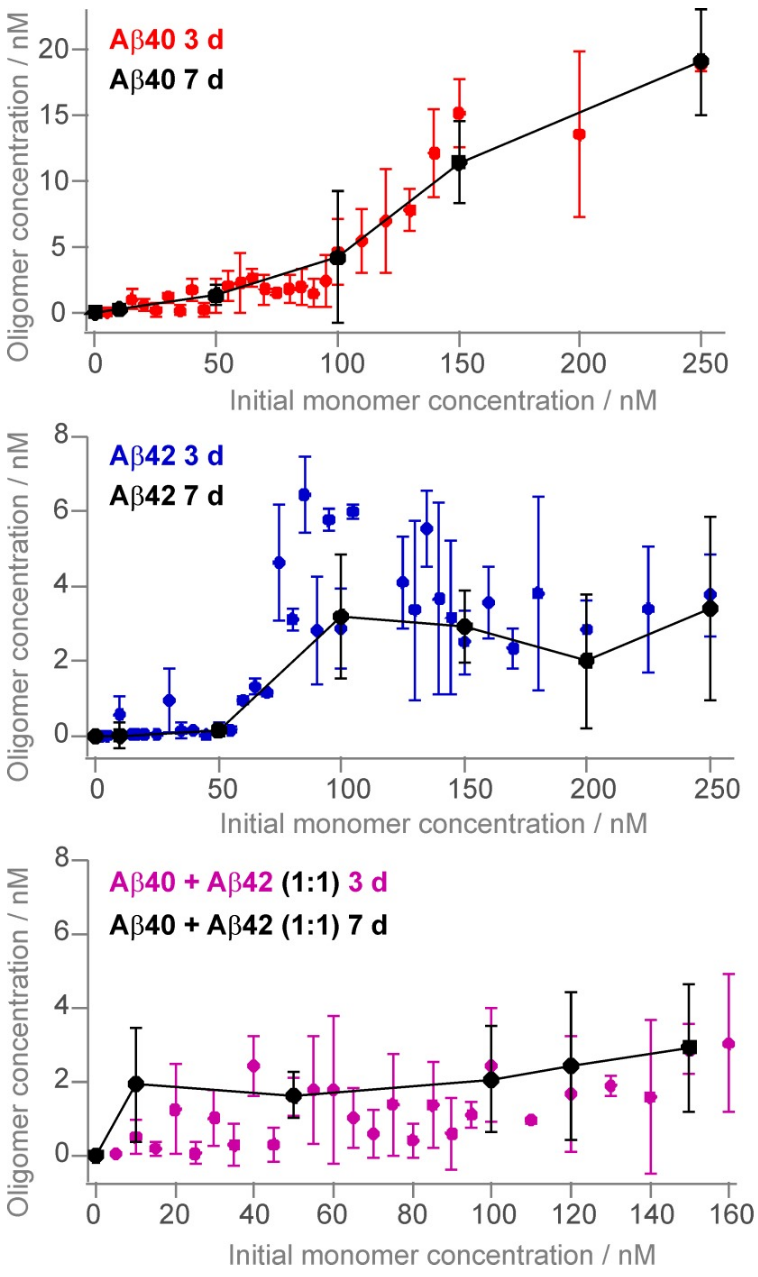

Figure S2. Oligomer concentrations as a function of the total initial monomer concentration in the aggregation reaction after 3 and 7 days of incubation. (Error bars SD, N (samples) = 3).

incident events.[3] Simultaneous events in both channels above the threshold (the AND criterion)[4] were selected and assigned to be due to Aβ oligomers. Any highly fluorescent events occupying multiple time-bins or consisting of more than 150 monomer units, resulting from dust or fibrillar aggregates, were excluded as described before,[1] making the measurements selective for oligomers. To account for any possible coincident events due to chance, the desynchronization approach was

used.[5] Time-bins in the blue channel were randomly re-numbered before the selection of simultaneous events in the two channels above the threshold. Using these outputs, the fraction of coincident events was estimated according to:

$$Q = \frac{C - E}{A + B + (C - E)} \tag{S1}$$

where A is the number of fluorescent bursts in the blue channel above the 15 photons bin^{-1} threshold, B is the number of fluorescent bursts in the red channel, C is the number of coincident events, and E is the number of chance-coincident events. The oligomer concentration was calculated as the fraction of coincident events (oligomers) multiplied by the starting total protein concentration.

To confirm that our TCCD experiments are probing the specific interaction between Aβ molecules, we performed control experiments using either 1:1 solutions of free fluorescent dyes, AlexaFluor 488 (Life Technologies) and AlexaFluor 647(Life Technologies); or 1:1 fluorescently-labeled immunoglobulins, AlexaFluor 488 Donkey Anti-Mouse InG (H+L) Antibody (Life Technologies) and AlexaFluor 647 Rabbit Anti-Mouse InG (H+L) Antibody (Life Technologies), keeping the same detection conditions as for the protein measurements. No coincidence other than chance-coincidence was observed in three separate measurements with either of these systems, excluding the possibility that positive results are due to noise or random association of fluorescent probes.

S2.2. CAC Measurements and Analysis

Data were acquired using the same single-molecule setup as for the above TCCD measurements. Single-color illumination was used, either 488 nm for the measurement of 488-labeled samples, or 633 nm for the measurements of 647-labeled samples. In the case of a dual-labeled DNA standard, data were acquired by exciting with one color, and subsequently with another color in the next recording. The measurements were stationary, with solutions placed on a glass coverslide and focusing the laser beam 10 μm into the solution. Data were acquired for 200 s with 1 ms time-bins (chosen according to the expected residence time in the confocal volume at these conditions[2]), 8000 bins per frame and a total of 25 frames.

Initially, a calibration curve was recorded using a dual-labeled 40 base pair DNA duplex, generated from double-HPLC purified complementary synthetic oligonucleotides (Atdbio) with the following sequences: AlexaFluor488-TAGTGTAACTTAAGCCTAGGATAAGAGCCAGTAATCGGTA,

and AlexaFluor647-TACCGATTACTGGCTCTTATCCTAGGCTTAAGTTACACTA.

The DNA duplex was formed by heating a 1:1 mixture of oligonucleotides (1 μM) to 95 °C followed by slow cooling to room temperature, analogous to previously used protocol.[2] Calibration was made with the reference to a triplicated measurement of the DNA standard, diluted in SSPE buffer, at a range of concentrations, which resulted in a linear increase in the number of bursts above a chosen threshold (10 counts bin^{-1}) with sample detection concentration (0-250 pM).

Subsequently, the supernatants collected from the fibrillar Aβ samples were diluted in SSPE (serial dilution) by a factor of 8×10^3 and measured as described. The number of bursts above the same threshold as for the standard (10 counts bin^{-1}) was counted and converted to the detection concentration, and subsequently to the bulk concentration using the dilution factor. The results are summarized in Table S1. Additionally, it was found in control measurements that comparable numbers of bursts were obtained in the samples after 3 and 7 d of incubation, confirming the equilibration past 3 d.

**Protein**	**Average number of Bursts ± SD (n=6)**	**[SM] ± SD ([SM]) / pM**	**[Bulk] ± SD ([Bulk]) / nM**
Aβ40-488	2370± 1316	12.7± 7.1	102 ± 57
Aβ40-647	2198± 1189	10.6± 5.7	85 ± 46
Aβ42-488	689± 127	3.65± 0.6	29.2 ± 4.8
Aβ42-647	673± 181	3.25± 0.85	26 ± 6.8

TABLE S1. Summary of CAC measurements. Average numbers of bursts are determined for supernatants above singly-labelled Aβ pellets. Using calibration to AF488 and 647 dual-labelled 40 base pair DNA strand, the numbers of bursts are converted to single-molecule concentrations ([SM]). The conversion to bulk sample concentrations ([Bulk]) is performed by multiplication of the single-molecule concentration by a corresponding sample dilution factor.

S3. MODELING OUTLINE

A streamlined statistical mechanical model was developed to describe the equilibrium oligomer size distributions studied in this work and to extract the free energy of oligomer growth by monomer addition. For the simple case of a single aggregating protein type, the equivalence of the proposed model to a well-established thermodynamic approach was demonstrated. Filamentous growth was assumed, such that oligomers are treated as one-dimensional chains. Nearest-neighbor interactions

were taken to dominate the partition function of each oligomer and were assumed independent of oligomer size. A single, size-independent equilibrium constant K thus describes the addition of a monomer to a j-mer to form a $(j+1)$-mer at temperature T in a reaction volume V, and can be used to derive the equilibrium concentration $f(j)$ of j-mers according to the relation

$$f(j) = \left(\frac{K}{c_0}\right)^{-1} \left[\left(\frac{K}{c_0}\right) \cdot f(1)\right]^j, \tag{S2}$$

where

$$K = \exp\left(-\frac{\Delta G^\circ}{RT}\right). \tag{S3}$$

Here, c_0 is the standard concentration (set by convention to 1 M), the temperature T is set to a standard 25 ^{o}C and R is the molar gas constant, leaving the standard free energy of elongation ΔG° as the sole free fitting parameter.

The equilibrium monomer concentration $f(1)$ entering Eq. (S2) is determined implicitly by conservation of the initial total monomeric protein concentation m_{tot}, as

$$\sum_{j=1}^{\infty} j \cdot f(j) = m_{\text{tot}}. \tag{S4}$$

The total oligomer concentration for a given initial concentration m_{tot} is described by taking the infinite sum of the distribution $f(j)$ from $j = 2$. The equivalent statistical mechanical formulation allows powerful generalization of these ideas. For example, the "invisible" single-color oligomers arising from the experimental methodology can be taken into account, allowing numerical comparison with experimentally-observed oligomer concentrations via least-squares fitting.

Given a fitted value for ΔG°, the dependence of oligomer concentration on oligomer length can be determined, and it is found that, at the concentrations studied, almost all oligomers are dimeric. Considering only monomers and dimers, the modelling approach can then be extended to describe the co-oligomerization of more than one monomer species. Such a technique provides a usable model that was fitted to Aβ40-Aβ42 co-oligomerization data to give the free energy of cross-elongation between the two species, and can also estimate how this free energy must in principle relate to the self-elongation energies in order for mixed oligomers to be formed. Full details can be found below.

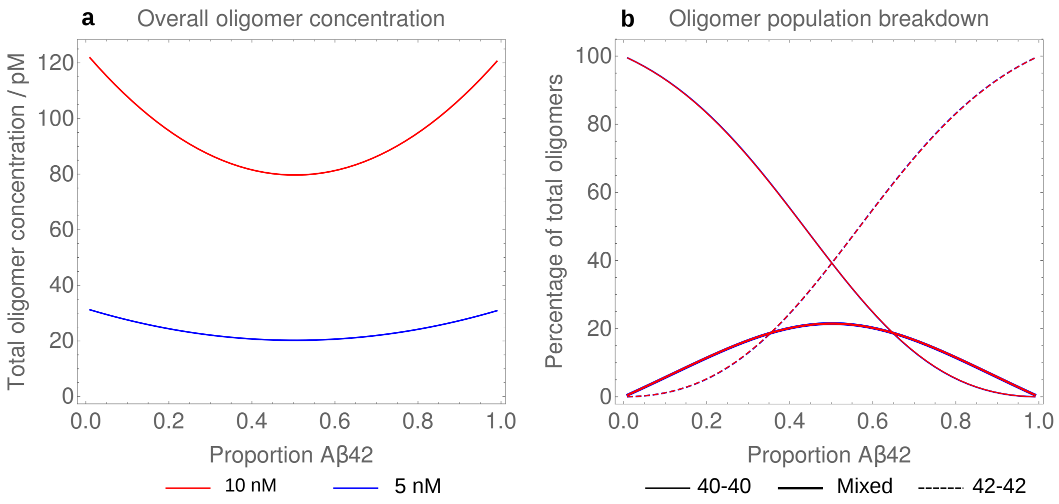

Figure S3. Simulation of Aβ40-Aβ42 co-oligomerization equilibrium behavior at 5 nM (blue) and 10 nM (red) total Aβ concentration for a range of Aβ40:Aβ42 ratios, using elongation free energies obtained from fitting to experimental data (see Figure 2 in main text). A clear difference is seen in total oligomer concentrations between the two monomer concentrations **(a)**, although the oligomer percentage breakdowns at both concentrations are virtually identical, with red and blue lines overlapping **(b)**.

S4. SIMULATIONS AT 5 nM AND 10 nM

Physiologically relevant concentrations of Aβ monomer are considered to be 1-10 nM. Figure S3 shows the results of simulations for total Aβ monomer concentrations of 5 nM and 10 nM, as described in Section S8. Figure 3 in the main text shows simulation results for 1 nM.

EXTENDED THEORY AND DERIVATION OF MODEL

S5. OVERVIEW

A simple statistical model was developed to describe the equilibrium size distribution of oligomers formed from aggregating monomer units. The model is derived from statistical mechanical principles for the aggregation of a single monomer type (Section S6), and its equivalence to a thermodynamic approach is demonstrated (Section S6.2). This comparison allows identification of an equilibrium constant, and the extraction of the free energy of monomer addition upon fitting the model to available data for the self-oligomerization of Aβ40 and Aβ42 (Sections S6.4 & S6.5). The statistical mechanical formulation of the model generalizes to include dimerization of multiple interacting monomer types (Section S7). Given that very few species larger than

dimers are present under conditions studied, this can be fitted to measurements of Aβ40 + Aβ42 co-oligomerization experiments (Section S7.3). Thus, the free energy of cross-elongation can also be estimated.

S6. SELF-OLIGOMERIZATION

S6.1. General formulation

A general expression for the concentration $f(j)$ of oligomers of size j in a system of volume V at temperature T can be found using the theoretical framework offered by the grand canonical ensemble. The grand canonical partition function is given by

$$\Xi(T,V,\mu) = \sum_{N}^{\infty} Q(T,V,N) e^{\beta N \mu} \tag{S5}$$

where $Q(T,V,N)$ denotes the canonical partition function of a system that contains N molecules, $\beta = 1/(k_B T)$ is the inverse temperature (k_B is the Boltzmann constant) and μ is the chemical potential of the monomers. We consider the formation of oligomeric clusters up to an arbitrary maximum size of M monomers. If we now assume that the clusters do not interact, we can then express the grand canonical partition function in terms of the cluster partition functions:

$$\Xi(T,V,\mu) = \sum_{N_1=0}^{\infty} \sum_{N_2=0}^{\infty} \cdots \sum_{N_M=0}^{\infty} \frac{\left(q_1(T,V)\, e^{\beta \mu}\right)^{N_1}}{N_1!} \frac{\left(q_2(T,V)\, e^{\beta 2 \mu}\right)^{N_2}}{N_2!} \cdots \frac{\left(q_M(T,V)\, e^{\beta M \mu}\right)^{N_M}}{N_M!}, \tag{S6}$$

where $q_j(T,V)$ is the canonical partition function of a j-mer. Eq. (S6) can be re-summed to yield

$$\Xi(T,V,\mu) = \exp\left(\sum_{j=1}^{M} q_j(T,V)\, e^{\beta j \mu} \right). \tag{S7}$$

The number of clusters of size n is then given by

$$N_j(T,V) = q_j(T,V)\, e^{\beta j \mu}. \tag{S8}$$

The concentrations of j-mers is given by $f(j) \equiv N_j(T,V)/(N_A V)$, where N_A is Avogadro's number. Re-writing $q_j(T,V)$ as $q(j)$, we obtain

$$f(j) = \frac{1}{N_A V} q(j)\, e^{\beta j \mu}. \tag{S9}$$

The partition functions $q(j)$ are given in general by a product of independent contributions from translational and internal degrees of freedom: $q(j) = q_{\text{trans}}(j)\, q_{\text{int}}(j)$. In particular, the translational partition function $q_{\text{trans}}(j)$ is proportional to the system volume, and so can be written as $q_{\text{trans}} = V/v_0(j)$, where $v_0(j)$ is a fundamental volume (in the gaseous phase, it is given by the cube of the thermal wavelength). In summary:

$$f(j) = \frac{1}{N_A\, v_0(j)}\, q_{\text{int}}(j)\, e^{\beta j \mu}. \tag{S10}$$

The chemical potential μ is set implicitly by conservation of the initial total monomeric protein concentration m_{tot}:

$$\sum_{j=1}^{\infty} j f(j) = -\frac{1}{N_A\, V}\frac{k_B\, T}{\Xi}\frac{\partial \Xi}{\partial \mu} = m_{\text{tot}}, \tag{S11}$$

where we have let $M \to \infty$ in Eq. (S7).

S6.2. Connection to thermodynamic approach

The jth equilibrium constant K_j, describing the association of a j-mer and a monomer to form a $(j+1)$-mer, is defined by

$$K_j = \frac{\frac{f(j+1)}{c_0}}{\frac{f(1)}{c_0} \cdot \frac{f(j)}{c_0}} \tag{S12}$$

$$= c_0\, \frac{f(j+1)}{f(1) \cdot f(j)}, \tag{S13}$$

where c_0 is the standard concentration. Eq. (S13) is solved recursively to give the equilibrium oligomer size distribution as

$$f(j) = \left(\prod_{i=1}^{j-1} \frac{K_i}{c_0} \right) f(1)^j, \tag{S14}$$

with $f(1)$ set implicitly by the conservation-of-mass requirement (Eq. (S11)). Comparison of Eq. (S14) to Eq. (S10) yields

$$f(1) = \frac{1}{N_A\, v_0(1)}\, q_{\text{int}}(1)\, e^{\beta \mu} \tag{S15}$$

and

$$K_j = N_A\, c_0 \cdot \frac{v_0(1)\, v_0(j)}{v_0(j+1)} \cdot \frac{q_{\rm int}(j+1)}{q_{\rm int}(1)\cdot q_{\rm int}(j)}. \tag{S16}$$

Finally, the standard free energy change $\Delta G^{\circ}(T, V, j) \equiv \Delta G^{\circ}(j)$ upon addition of a single monomer to an oligomer of size j is obtained from Eq. (S16) using the relation

$$\Delta G^{\circ}(j) = -R\,T \ln K_j, \tag{S17}$$

where the values chosen for T and c_0 determine the standard conditions.

S6.3. Oligomer partition function

We assume that the binding free energy associated with the nearest-neighbor interactions between constituent monomers is equal to ε, irrespective of the size of the oligomer, such that

$$\frac{q_{\rm int}(j+1)}{q_{\rm int}(1)\cdot q_{\rm int}(j)} = e^{-\beta\varepsilon}, \tag{S18}$$

and that the free energy of a j-mer, excluding translational entropic contiributions, is thus given by

$$U(j) = (j-1)\,\varepsilon \tag{S19}$$

as a j-mer has $j-1$ nearest-neighbor interactions. The internal oligomer partition function $q_{\rm int}(j)$ is therefore approximated as

$$q_{\rm int}(j) \approx e^{-\beta\,\varepsilon\,(j-1)}. \tag{S20}$$

Furthermore, we assume that the volume-dependent translational entropic contribution from $q_{\rm trans}(j)$ is independent of oligomer size, such that $v_0(j) \equiv v_0$ is constant. Together, these two simplifications are equivalent to postulating size-independent aggregation and disaggregation rates in a filamentous growth model, as made clear by the now size-independent equilibrium constant

$$K_j \approx N_A\, c_0\, v_0\, e^{-\beta\,\varepsilon} \equiv K \tag{S21}$$

from Eq. (S16), and the resulting expression for the oligomer size distribution from Eq. (S14):

$$f(j) = \left(\frac{K}{c_0}\right)^{j-1} f(1)^j = \frac{1}{N_A\, v_0}\, e^{-\beta\,\varepsilon\,(j-1)}\, e^{\beta\, j\, \mu}. \tag{S22}$$

The now size-independent standard free energy change ΔG° upon addition of a monomer to an oligomer is found from Eq. (S17) to be

$$\Delta G^\circ = \varepsilon - R\,T \ln\left(N_A\, c_0\, v_0\right). \tag{S23}$$

Under the simplifying assumptions described in this section, we can now evaluate Eq. (S7) analytically in the limit $M \to \infty$ to give

$$\Xi(T, V, \mu) = \exp\left(\frac{V}{v_0} \frac{e^{\beta\mu}}{1 - e^{-\beta\varepsilon} e^{\beta\mu}}\right), \tag{S24}$$

and Eq. (S11) can be evaluated similarly to give

$$m_{\mathrm{tot}} = \frac{1}{N_A\, v_0} \frac{e^{\beta\,\mu}}{\left(1 - e^{\beta\,\mu} e^{-\beta\,\varepsilon}\right)^2}. \tag{S25}$$

By combining this expression with Eqs. (S10) and (S20), an implicit expression for ε can be found if the oligomer size distribution $f(j)$ is known:

$$m_{\mathrm{tot}}\, v_0\, N_A = \frac{\left[N_A\, v_0\, f(j)\right]^{1/j} e^{\beta \frac{j-1}{j} \varepsilon}}{\left\{\left[N_A\, v_0\, f(j)\right]^{1/j} e^{-\beta \frac{\varepsilon}{j}}\right\}^2}, \tag{S26}$$

where we have set $q'(j) = 1$. Writing $\varepsilon = \Delta G^\circ + R\,T \ln\left(N_A\, c_0\, v_0\right)$, as described by Eq. (S23), ΔG° emerges as being independent of v_0, as the term $v_0\, N_A$ drops out from both sides of Eq. (S26). This result can be understood by interpreting the term $1/(N_A\, v_0)$ as a concentration and by noticing that ε is the free energy ΔG measured at standard concentration $c = 1/(N_A\, v_0)$:

$$\Delta G = \Delta G^\circ + R\,T \ln\left(\frac{c_0}{c}\right). \tag{S27}$$

Hence, we choose $v_0 = 1/(N_A\, c_0)$ for convenience, such that $\Delta G^\circ = \Delta G$, where standard conditions are chosen as $T = 25$ °C and $c_0 = 1$ M.

S6.4. Correcting for experimental observations

In each experiment, half of the monomer species are labeled "red", and the other half "blue". Only oligomers which contain at least one red monomer and at least one blue monomer are accounted for; all single-color species detected are branded free monomer molecules by the experimental procedure, and the two labels are assumed to have no differing effects besides introducing a degree of distinguishability between particles.

Eq. (S10) is thus modified to take into account the two colors:

$$f(j) = \sum_{j_1=0}^{j} f(j_1, j-j_1) = \frac{1}{N_A\, v_0}\, e^{-\beta\,\varepsilon(j-1)} e^{\beta\, j\, \mu} \sum_{j_1=0}^{j} \binom{j}{j_1} = \frac{2^j}{N_A\, v_0}\, e^{-\beta\,\varepsilon(j-1)} e^{\beta\, j\, \mu}, \tag{S28}$$

where $f(j_1, j_2)$ denotes the concentration of oligomers of size $j = j_1 + j_2$ monomers, of which j_1 are red and j_2 are blue. Eq. (S24) is similarly modified to give

$$\begin{aligned} \Xi(T, V, \mu) &= \exp\left(\sum_{j=1}^{M} \sum_{j_1=0}^{j} q(j_1, j-j_1)\, e^{\beta j \mu} \right) \\ &= \exp\left(\sum_{j=1}^{M} \frac{2^j\, V}{v_0} e^{-\beta\,\varepsilon(j-1)} e^{\beta\, j\, \mu} \right) \\ &= \exp\left(\frac{V}{v_0} \frac{2e^{\beta\mu}}{1 - 2e^{-\beta\varepsilon} e^{\beta\mu}} \right), \end{aligned} \tag{S29}$$

where $M \to \infty$ in the last line.

Terms in Eq. (S28) are then grouped according to the experimental constraints, to give an apparent size distribution $f_{\mathrm{exp}}(j)$ that can be compared directly with experimental data:

$$f_{\mathrm{exp}}(j) = \begin{cases} \sum_{j_1=1}^{\infty} f(j_1, 0) + \sum_{j_2=1}^{\infty} f(0, j_2) & j = 1 \\ \sum_{j_1=1}^{j-1} f(j_1, j-j_1) & j > 1 \end{cases}. \tag{S30}$$

Finally, the conservation-of-mass condition Eq. (S11) for determination of μ is modified using Eq. (S29) to conserve initial system mass for red monomers (and by symmetry, also for blue monomers, which are associated with a numerically identical chemical potential):

$$\sum_{j=1}^{\infty}\sum_{j_1=0}^{j} j_1 \cdot f(j_1, j-j_1) = -\frac{1}{2}\frac{1}{N_A V}\frac{k_B T}{\Xi}\frac{\partial \Xi}{\partial \mu}$$
$$= \frac{1}{N_A v_0}\frac{e^{\beta\mu}}{(1-2e^{\beta\mu}e^{-\beta\varepsilon})^2} \quad \text{(S31)}$$
$$= \frac{1}{2}m_{\text{tot}}.$$

S6.5. Fitting procedure

Eq. (S30) can be fitted directly via least-squares methods to experimental measurements of equilibrium j-mer concentrations, with ε as the sole fitting parameter.

Oligomer size distributions are difficult to measure reliably. Thus, the function $F_{\text{exp}}(m_{\text{tot}}) = \sum_{j=2}^{\infty} f_{\text{exp}}(j)$ is usually fitted instead to measurements of total equilibrium oligomer concentrations for a range of initial monomer concentrations m_{tot} (the dependence of F on m_{tot}, implicit in μ, has been made explicit here). At each step, μ is evaluated numerically from the implicit analytical conservation-of-mass condition Eq. (S31), and the upper limit of the sums involved are set so that the mass contribution neglected from larger species introduces an error of less than 1 part in 10^6; an upper limit of $j = 40$ was used in this study, proving easily sufficient. Errors in ε are estimated by manually varying the parameter until the resulting curve no longer falls within the majority of the error bars on the datapoints.

S7. CO-OLIGOMERIZATION

The approach detailed in Section S6 can be generalized to consider the co-oligomerization of more than one species to form dimers.

S7.1. Exact solution

The grand canonical partition function for a co-oligomerizing ensemble of 2 different monomer types, labelled a and b, up to a maximum oligomer size of 2, is given as a direct generalization of Eq. (S6):

$$\Xi(T,V,\vec{\mu}) = \sum_{N_a=0}^{\infty}\sum_{N_b=0}^{\infty}\sum_{N_{aa}=0}^{\infty}\sum_{N_{bb}=0}^{\infty}\sum_{N_{ab}=0}^{\infty} \frac{\left(q_a(T,V)\, e^{\beta\mu_a}\right)^{N_a}}{N_a!} \frac{\left(q_b(T,V)\, e^{\beta\mu_b}\right)^{N_b}}{N_b!} \\ \frac{\left(q_{aa}(T,V)\, e^{\beta 2\mu_a}\right)^{N_{aa}}}{N_{aa}!} \frac{\left(q_{bb}(T,V)\, e^{\beta 2\mu_b}\right)^{N_{bb}}}{N_{bb}!} \frac{\left(q_{ab}(T,V)\, e^{\beta(\mu_a+\mu_b)}\right)^{N_{ab}}}{N_{ab}!} \tag{S32}$$

This becomes, as a direct generalization of Eq. (S7):

$$\Xi(T,V,\vec{\mu}) = \exp\left(q_a\, e^{\beta\mu_a} + q_b\, e^{\beta\mu_b} + q_{aa}\, e^{2\beta\mu_a} + q_{bb}\, e^{2\beta\mu_b} + q_{ab}\, e^{\beta(\mu_a+\mu_b)}\right), \tag{S33}$$

where e.g. q_{ab} gives the canonical partition function for a dimer, and $f(ab)$ gives their concentration. The chemical potential μ_i of monomer type i is defined for corresponding initial type i total monomeric protein concentration m^i_{tot} by

$$\begin{aligned} f(a) + f(ab) + 2f(aa) &= -\frac{1}{N_A\,V}\frac{k_B\,T}{\Xi}\frac{\partial\,\Xi}{\partial\,\mu_a} = m^a_{\text{tot}}, \\ f(b) + f(ab) + 2f(bb) &= -\frac{1}{N_A\,V}\frac{k_B\,T}{\Xi}\frac{\partial\,\Xi}{\partial\,\mu_b} = m^b_{\text{tot}}. \end{aligned} \tag{S34}$$

If nearest-neighbor interactions dominate, then we can write $q_{ij} = \frac{V}{v_0}\exp(-\beta\epsilon_{ij})$ in analogy with the single-species case.

S7.2. Application to present work

The single-species model, in conjunction with the fitted single-species values for oligomerization free energy, can be used to calculate the proportion of oligomers in the single-species experiments that are dimers. At the range of concentrations explored in this study, this proportion ranges from 90-100%. Total observed oligomer concentrations are lower in the 2-species experiments, implying cross-oligomerization to be less favorable than single-species oligomerization, and therefore that the proportion of oligomers larger than dimers is even lower than in the single species experiments. Under such conditions it is therefore appropriate to fit the 2-species oligomer data to our dimer-only model derived to estimate ϵ_{40-42}. Error inherent in the model due to neglection of larger oligomers is expected to be smaller than experimental error.

S7.3. Correcting for experimental observations and fitting

For two species, one labeled red (type 1) and the other blue (type 2), we can fit the expression for $f(ab)$ directly to experimental measurements, where $f(ab)$ is explicitly given by

$$f(ab) = \frac{V}{v_0} e^{-\beta\epsilon_{ab}} e^{\beta(\mu_a+\mu_b)} \tag{S35}$$

The fitting procedure now involves evaluating both chemical potentials at each stage numerically from implicit algebraic expressions, obtained via Eq. (S34). The sole fitting parameter is $\varepsilon_{1,2}$, once $\varepsilon_{1,1}$ and $\varepsilon_{2,2}$ are established from fits to self-oligomerization data sets. The same standard value for v_0 is used for all fits. Elongation free energies can be found for different concentrations as before, using Eq. (S27), and errors are estimated as before. All experiments were carried out at $T = 37$ °C, and this is used to calculate an appropriate value of β for the fitting.

S8. SIMULATING DIFFERENT CONCENTRATIONS AND RATIOS

Once all elongation free energies have been found from fitting, the expressions above can be used to simulate co-oligomerization equilibrium concentrations of different species for a range of concentrations of each monomer type (provided we remain within the range of concentrations at which dimers dominate), with the chemical potentials set in all cases by Eq. (S34). The composition distribution $f(ij)$ can be calculated explicitly, and the total oligomer concentrations can be predicted from their sum. The results can be seen in Fig.S3, and in Fig.3 of the main text. Finally, it is of interest to consider the maximum predicted total equilibrium oligomer concentration. This is capped because the equilibrium monomer concentration is limited by the CAC. Using the CAC as an accurate estimate for the total concentration of abeta in monomers and in oligomers, we can calculate the maximum possible total equilibrium oligomer concentration to be approximately 44nM. The approximation that oligomers are predominantly dimers is at its least accurate at this upper limit of the monomer concentration; however, we estimate that the error introduced to the total oligomer concentration by this approximation is no more than 5%. To arrive at this error estimate, we simulated a single-species system at the same total monomer concentration, using the single-species oligomerisation free energy of A40, and discovered that when the permitted oligomer species were restricted to dimers the calculated total oligomer concentration is 95% of the total

oligomer concentration obtained when all oligomer lengths are permitted.

---

[1] Horrocks, M. H. *et al.* Fast Flow Microfluidics and Single-Molecule Fluorescence for the Rapid Characterization of alpha-Synuclein Oligomers. *Anal Chem*, doi:10.1021/acs.analchem.5b01811 (2015).

[2] Horrocks, M. H. *et al.* Single molecule fluorescence under conditions of fast flow. *Anal Chem* **84**, 179-185, doi:10.1021/ac202313d (2012).

[3] Clarke, R., Orte, A. & Klenerman, D. Optimized threshold selection for single-molecule two-color fluorescence coincidence spectroscopy. *Analytical Chemistry* **79**, 2771-2777, doi:10.1021/ac062188w (2007).

[4] Ying, L., Wallace, M., Balasubramanian, S. & Klenerman, D. Ratiometric analysis of single-molecule fluorescence resonance energy transfer using logical combinations of threshold criteria: A study of 12-mer DNA. *Journal of Physical Chemistry B* **104**, 5171-5178, doi:10.1021/jp993914k (2000).

[5] Orte, A., Clarke, R., Balasubramanian, S. & Klenerman, D. Determination of the fraction and stoichiometry of femtomolar levels of biomolecular complexes in an excess of monomer using single-molecule, two-color coincidence detection. *Analytical Chemistry* **78**, 7707-7715, doi:10.1021/ac061122y (2006).

[6] Baxter, R. J. *Exactly solved models in statistical mechanics* **1982**. Academic Press Inc., London.

[7] Knowles, T. P. J.; Waudby, C. A.; Devlin, G. L.; Cohen, S. I. A.; Aguzzi A.; Vendruscolo, M.; Terentjev, E. M.; Welland, M. E.; Dobson, C. M. An Analytical Solution to the Kinetics of Breakable Filament Assembly *Science* **326**, 1533 (2009).
